# Active Transportation Safety Features around Schools in Canada

**DOI:** 10.3390/ijerph10115711

**Published:** 2013-10-31

**Authors:** Bryn Pinkerton, Andrei Rosu, Ian Janssen, William Pickett

**Affiliations:** 1 Department of Public Health Sciences, Queen’s University, Kingston, ON K7L 3N6, Canada; E-Mails: bryn.pinkerton@mail.mcgill.ca (B.P.); ian.janssen@queensu.ca (I.J.); 2 Departments of Biology and International Development Studies, McGill University, Montreal, QC H3A 0G4, Canada; 3 School of Kinesiology and Health Studies, Queen’s University, Kingston, ON K7L 3N6, Canada; E-Mail: rosua@queensu.ca; 4 Department of Emergency Medicine, Queen’s University, Kingston, ON K7L 2V7, Canada

**Keywords:** cyclist, environment, injury, pedestrian, policy, road, safety, schools, traffic

## Abstract

The purpose of this study was to describe the presence and quality of active transportation safety features in Canadian school environments that relate to pedestrian and bicycle safety. Variations in these features and associated traffic concerns as perceived by school administrators were examined by geographic status and school type. The study was based on schools that participated in 2009/2010 Health Behaviour in School-aged Children (HBSC) survey. ArcGIS software version 10 and Google Earth were used to assess the presence and quality of ten different active transportation safety features. Findings suggest that there are crosswalks and good sidewalk coverage in the environments surrounding most Canadian schools, but a dearth of bicycle lanes and other traffic calming measures (e.g., speed bumps, traffic chokers). Significant urban/rural inequities exist with a greater prevalence of sidewalk coverage, crosswalks, traffic medians, and speed bumps in urban areas. With the exception of bicycle lanes, the active transportation safety features that were present were generally rated as high quality. Traffic was more of a concern to administrators in urban areas. This study provides novel information about active transportation safety features in Canadian school environments. This information could help guide public health efforts aimed at increasing active transportation levels while simultaneously decreasing active transportation injuries.

## 1. Introduction

Active transportation has been defined as walking, cycling, or using other forms of physical activity for the purpose of transportation [1]. It allows children and youth to participate in physical activity in a functional context of getting to school and other destinations. Streets, roads and other man-made surroundings can have an influence on active transportation behaviours, especially among children [2,3]. To illustrate, the presence of pedestrian and cyclist infrastructure (sidewalks, crosswalks, bicycle lanes) is associated with higher levels of active transportation in industrialized countries [4,5]. Lack of road safety features, traffic concerns, and perception of danger are factors that may hinder such transportation [6,7,8,9]. Furthermore, for those young people who engage in active transportation, the absence of such safety features and excessive traffic can lead to injury. Child pedestrians account for 5%–10% of road traffic deaths in high-income countries and 30%–40% in middle to low-income countries [10]. In addition, cyclists constitute 3%–15% of reported traffic injuries, and up to 8% of traffic deaths internationally [10], with a substantial burden of such injuries reported for Canada [11].

In addition to examining the burden of the school pedestrian and cyclist road safety issues [11,12,13,14], existing research has emphasized the efficacy of traffic calming schemes and other safety features [9]. Such initiatives suggest that their installation and maintenance enhance safety. A study conducted in the United Kingdom reported significant decreases in child pedestrian injuries rates (2.14 per 1,000 population) in a city after the implementation of multiple traffic calming features [11]. Yet, studies dedicated to describing the active transportation safety environment surrounding schools are scarce, with some exceptions from international settings [15,16,17]. Results vary according to the country’s economic status, with more advanced infrastructure generally located in high-income countries. There has been much attention given to the presence of crosswalks and sidewalks, as well as the visibility of speed reduction signs in this literature [15,16,17]. Canadian studies of such features are lacking. Studies have also not considered other features that may contribute to safety like bicycle lanes, speed bumps, traffic medians, or traffic chokers especially in Canadian settings.

Geographic variations in active transportation injury rates have been observed. For instance, child cyclists in rural environments are approximately twice as likely (RR: 2.30) to be injured from a motor vehicle traffic collision than are child cyclists in urban environments [18]. Geographic variations in the active transportation road and other safety features surrounding schools are less clear, especially in Canada.

In the current study, we therefore describe the presence and quality of active transportation safety features in environments surrounding Canadian schools. We also identify associated traffic concerns for these school neighborhoods, as perceived by school administrators, as well as variations in these features and concerns by urban-rural geography and school type (primary *vs*. mixed *vs*. secondary schools). The study base was Canadian schools that participated in the 2009/2010 Health Behaviour in School-aged Children (HBSC) survey. Our hope was that this foundational information would inform policy development initiatives for both physical activity initiatives and also injury prevention in Canada.

## 2. Methods

### 2.1. Survey Overview

HBSC is a cross-national study that examines health and its contextual determinants in 11–15 year olds [19]. In 2009–2010, 43 different countries and regions in Europe, North America and the Middle East participated; this study is based solely on the Canadian data. The 2010 Canadian HBSC contained three components: (1) a health survey completed by 26,078 grade 6–10 students from 436 schools; (2) geographic information system (GIS) measures of the characteristics of roads and sidewalks and associated safety features surrounding all 436 schools; and (3) an administrator survey completed by a principal or designate at 411 of the 436 participating schools. The present study is based on the latter two data sources.

Our analyses here involved schools in eight provinces and the three territories (all but Prince Edward Island and New Brunswick participated; over-samples were administered in certain provinces (British Columbia, Alberta, Saskatchewan and Newfoundland and Labrador) and a census of all young people attending school was attempted in the three northern territories (Yukon Territory, North West Territories, and Nunavut). The national sample of schools was stratified by province/territory, type of school board (public *vs*. separate), urban-rural geographic status, school population size, and language of instruction (French *vs*. English) with standardized population weights generated for individual students to account for the oversampling and stratification criteria. Children from private schools, home school situations, Native reserves, street youth, incarcerated youth, and youth not providing informed consent (explicit or implicit, as per local school board customs) were excluded. The survey was administered during the months of November 2009 through May 2010. Response rates were 11/13 (84.6%) at the province/territorial level, 436/765 (57.0%) at the level of schools, and 26,078/33,868 (77.0%) at the individual student level. At the school board and school levels, sampling was done with replacement.

### 2.2. GIS Measures

We used ArcGIS version 10 (Esri, Redlands, CA, USA, 2010) and Google Earth software to assess the presence of environmental safety features relevant for active transportation, as well as the quality of some of these features. In order to obtain a comprehensive assessment of these environments, GIS measures were obtained for: (1) all roads within a 400 metre Eucladian distance radius around each school; (2) all roads that immediately surrounded the school grounds; or (3) portions of the road immediately in front of each school’s main entrance. Google Earth street view was used to identify each school’s main entrance. In extensive pilot work with 30 schools, the 400 metre buffer was found to capture known features of the school transportation environment that could lead to child pedestrian or cycling injury on a consistent basis. It was assumed that all children who were engaged in active transportation to school would have to commute through this buffer. Finally, assessment of these three facets of the school active transportation environments was feasible given study resources.

**Figure 1:**
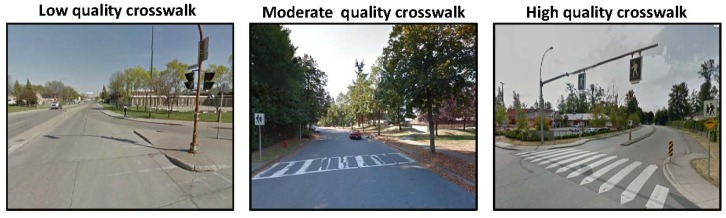
Google Street View images of crosswalks.

**Figure 2:**
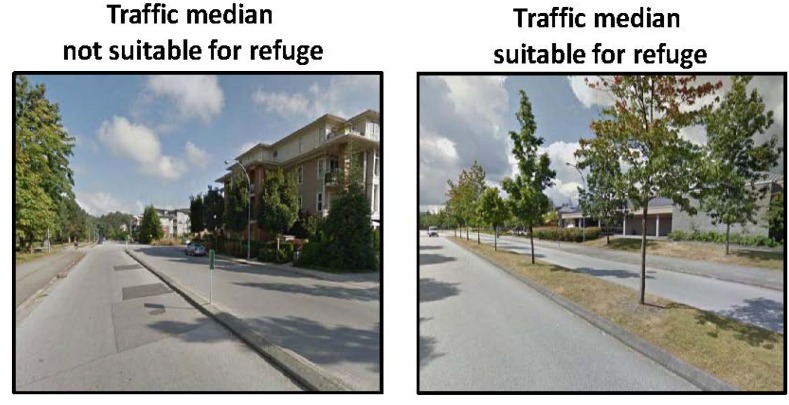
Google Street View images of traffic medians.

**Figure 3:**
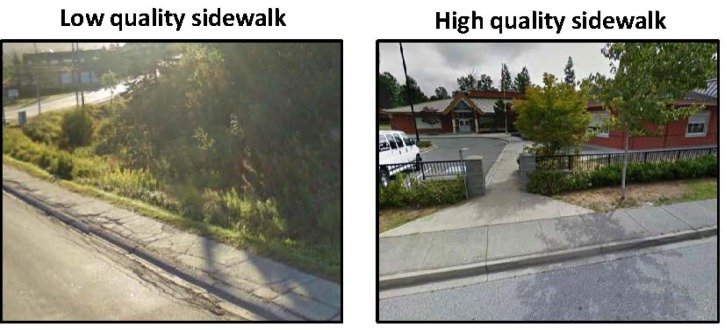
Google Street View images of sidewalk quality.

**Figure 4:**
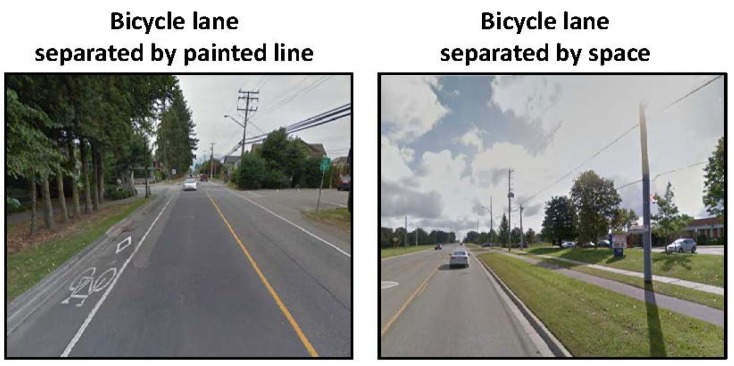
Google Street View images of bicycle lanes.

Google Earth software and (where not available) associated satellite imagery provide proxy measures for environmental scans that identify and quantify specific features of road and other transportation environments [20]. Figure 1, Figure 2, Figure 3 and Figure 4 provide examples of the types of images that were available for the three assessments. Following validation checks for consistency of data abstraction, the lead investigator (BP) systematically examined virtual images for all sentinel street and road environments for each school, and recorded observations for key variables on a data abstraction sheet. These were then summarized for each school. Of note, data available from Google Earth are still limited in Canadian school environments located in highly rural and remote areas. Satellite images were used as a replacement when street images were not available, and in some communities no clear images were available from either geographic source. The numbers of schools with available data for each of the measures are provided in our summary tables.

*Type of Road*: These measures were obtained for all roads within the 400 m radius of each school using the CanMap Route Logistics street file in ArcGIS. We determined the percentage of the total road distance that was comprised of the following road types: “*expressways*”, “*highways*”, “*major municipal roads*”, or “*local municipal roads*”.

*Distance Across Road*: The distance across the road directly in front of the main entrance to each school was measured using the ruler tool in Google Earth. Schools were placed into the following groups: “*≤10 metres*”, “*11–20 metres*”, or “*≥21 metres*”.

*Speed Reduction* *Sign*: We assessed whether there was at least one speed reduction sign (“*yes*” or “*no*”) beside the roads immediately surrounded each school’s grounds using Google Earth street view images.

*Sidewalk Coverage*: This measure was based on the roads immediately surrounding the school grounds. Extensive details are published elsewhere [21]. Briefly, using the CanMap Route Logistics street network in ArcGIS, we extracted the roads segments for these roads. All road segments were examined using satellite and street view imagery in Google Earth, and segments that did not have a sidewalk on either side were removed, and the remaining road segments were exported to ArcGIS where we calculated the percentage of the total road distance with sidewalk coverage. Schools were grouped as having 100% sidewalk coverage or <100% coverage.

*Presence of Road Safety Features*: Google Earth street view was used to count the number of the following safety features on the roads immediately surrounded each school’s grounds: traffic medians (e.g., solid object in the central portion of a road that separates opposing traffic), speed bumps, traffic chokers (e.g., narrowing at outside of road typically achieved by widening the sidewalk), bicycle lanes, crosswalks located at intersections, and crosswalks that were not at an intersection. Schools were grouped as either having or not having each of these features (“*yes*” or “*no*”).

*Quality of Safety Features*: Qualitative assessments were performed systematically for all safety features located on the roads immediately surrounded each school’s grounds. If more than one safety feature was present for a given school, an average quality score was determined and used for analytic purposes. Reliability tests for the Google Earth measures were conducted to determine inter and intra-rater agreement. Thirty schools with available Google Earth street view were randomly selected and the initial rater completed the measures a second time while a second rater completed these measures a single time. The intra-rater agreement between the ratings for the different qualitative measures was excellent (range 89.7% to 95.6%, with an average of 93.6%). The inter-rater agreement ratings were similarly excellent (88.7% to 95.6%, with an average of 92.1%).

Crosswalks located at intersections and non-intersections were assessed as being of a “*high quality*” (contained a brightly painted marking pattern), “*moderate quality*” (faded painted marking pattern), or “*low quality*” (very faded and difficult to identify painted marking pattern) (see Figure 1) [22]. Traffic medians were categorized as being “*suitable for refuge*” (median provided adequate space for pedestrian to stand on) or “*not suitable for refuge*” (median considered unsafe for pedestrian to stand on) (Figure 2). Sidewalks were categorized as being of a “*high quality*” (minimal cracks and surface defects) or “*low quality*” (frequent large cracks and other surface defects) (Figure 3). Bicycle lanes were categorized as being “*separated from automobile traffic by a painted line*” or “*separated from automobile traffic by physical barrier or space*” such as a curb or boulevard (Figure 4).

### 2.3. Administrator Survey Measures of Traffic Problems

In the HBSC administrator survey, principals were asked to classify the automobile traffic in their school neighbourhood as being: *not being a problem*, *a minor problem*, *a moderate problem*, *or a major problem*. Relevant administrator survey data were not available for 37 schools.

### 2.4. Statistical Analysis

Descriptive results are presented for all schools and by key contextual factors. The latter included urban-rural geographic status, estimated based upon Statistics Canada criteria [23]: rural (population <1,000), small population centre (1,000 to 29,999), medium population centre (30,000 to 99,999) and urban centre (>100,000). Schools were also categorized according to the student population served: primary (kindergarten to grade 8), secondary (grades 9 to 12) and mixed (containing grades covered in both primary and secondary schools). Chi-square tests were used to determine the significance of differences between measures according to urban/rural status and grade served by the school. Similar analyses were used to relate administrators’ perceptions of traffic to road types and other safety features. Statistical analyses were conducted using SPSS (IBM Corp., Armonk, NY, USA) and SAS (SAS Inc., Cary, NC, USA) statistical software.

## 3. Results

Table 1 provides a description of the pedestrian and cycling safety features that are present in the environments surrounding Canadian schools. The majority (85%) of roads located near schools were local roads, with only a small percentage being highways or expressways. Distance across the roads directly in front of the main school entrance averaged 13 m (range 5 to 30 m), and this was highest in urban (average 14 m) *vs*. compared with other school locations (13 m, 13 m, and 12 m for medium, small, and rural locations, respectively). There was no meaningful difference in this distance observed by school type.

Approximately 63% of the schools had a speed reduction sign posted on the roads surrounding to the school property and 85% of the schools had complete sidewalk coverage. Most schools had at least one crosswalk nearby, particularly at intersections (81%). Other traffic calming measures, such as traffic chokers (5%) and speed bumps (5%), were less common. Only 10% of schools had a designated bicycle lane nearby.

Significant urban/rural gradients were observed for four of the 10 safety feature measures (sidewalk coverage, crosswalks, traffic medians, speed bumps). These measures were all more common in medium sized communities and urban areas, with traffic medians and speed bumps being nearly absent in rural and small areas. Significant differences were also found by student population served. Secondary schools were more likely to have sidewalk coverage on all of the roads surrounding the school grounds and large roads (>21 m) were more frequent in their environments as well. No geographic differences were observed for the proportion of intersections with a marked crosswalk.

The quality of the safety features that were present are described in Table 2. A sizeable proportion of the crosswalks, whether located at intersections (31%) or not at intersections (18%), were of a low quality. Conversely, only 1% of the sidewalks were of a low quality. When traffic medians were present, 89% of the time they were rated as suitable for pedestrian refuge. The majority (81%) of the 32 bicycle lanes there were identified were separate from automobile traffic by a painted line rather than by a physical barrier or space. Most of the quality measures did not vary significantly by urban/rural location or type of school.

As shown in Table 3, 52% of the school administrators felt that heavy traffic was not a problem in their school’s neighbourhood, although this proportion decreased when looking at schools in large urban centers. Heavy traffic was perceived to be a moderate or major problem for 29% of the schools in urban centres. The administrators’ perceptions of major traffic problems were associated with strong and statistically significant increases in the presence of major roads (*p* < 0.002), increased distances across the roads in front of main school entrances (*p* < 0.001), and the absence of traffic medians (*p* < 0.001) (data not shown).

## 4. Discussion and Conclusions

This study described active transportation safety features and perceived traffic concerns around Canadian schools. Our hope was this foundational information would inform public health policy in Canada related to both the promotion of physical activity, as well as injury prevention among populations of young people. Our primary findings were that conventional road features that promote active transportation (e.g., sidewalks and crosswalks) were commonly present around Canadian schools, and generally of good quality. However, more progressive safety features (e.g., bicycle lanes, speed bumps, traffic medians, and chokers) were less prevalent in these Canadian school environments. More specifically, our assessments suggested that: (1) existing roads generally have some pedestrian infrastructure but are lacking cycling-specific infrastructure; (2) there is an inequity in the presence of certain safety features around schools according to geographic (urban/rural) status; and (3) traffic is more of a more obvious concern to responding administrators in urban areas.

**Table 1:** Presence of pedestrian and cycling safety features in environments surrounding Canadian schools.

	Total	Urban/Rural Location of School					Type of School			
		Rural	Small	Medium	Urban	*p*-Value	Primary	Mixed	Secondary	*p*-Value
Type of road (No. of schools with GIS)	436	59	187	67	123		193	120	123	
Expressway, average%	1.2	0.1	0.9	1.3	3.1	0.16	1.7	2.3	1.2	0.80
Highway, average%	6.0	11.3	6.5	4.8	2.4		5.1	4.5	5.1	
Major road, average%	8.0	0.3	6.1	9.1	13.8		9.1	9.6	9.3	
Local road, average%	84.8	88.3	86.5	84.8	80.6		84.0	83.7	84.4	
Distance across road (No. of schools with Google Earth)	372	35	150	65	122		170	98	104	
Minimum (≤10 metres), %	29.8	40.0	34.0	30.8	21.3	0.18	25.9	35.7	30.8	0.03
Average (11 to 20 metres), %	63.2	54.3	61.3	61.5	68.9		70.6	56.1	57.7	
Maximum (≥21 metres), %	7	5.7	4.7	7.7	9.8		3.5	8.2	11.5	
Speed reduction sign near school (No. of schools with Google Earth)	339	23	129	64	123		153	78	108	
Yes, %	63.4	52.2	65.9	68.8	60.2	0.40	64.7	62.8	62.0	0.90
Complete sidewalk coverage (No. of schools with Google Earth)	404	44	173	64	123		181	110	113	
Yes, %	85.1	59.1	76.9	98.4	99.2	<0.001	86.7	73.7	93.8	<0.001
Presence of safety features (No. of schools with Google Earth)	326	21	123	60	122		147	73	106	
Crosswalks not at intersections, %	27.9	9.5	31.7	28.3	27.0	0.20	32.7	18.4	10.9	0.20
Crosswalks at intersections, %	81.0	0	75.6	93.3	89.3	<0.001	84.4	80.2	75.3	0.28
Proportions of intersections with crosswalks, %	58.5	57.1	57.1	61.4	58.3	0.94	60.6	56.9	56.4	0.82
Traffic median present, %	30.0	4.8	22.0	35.0	40.2	<0.001	23.8	34.2	35.8	0.13
Traffic choker present, %	5.5	0	4.1	5.0	9.0	0.15	5.5	0.9	6.6	0.78
Speed bump present, %	5.5	0	1.6	10.0	8.2	0.03	6.1	6.8	3.8	0.62
Bicycle lane present, %	10.1	0.2	6.2	7.5	10.7	0.25	8.8	6.5	8.5	0.28

**Table 2:** Quality of pedestrian and cycling safety features in environments surrounding Canadian schools.

	Total	Urban/Rural Location of School					Type of School			
		Rural	Small	Medium	Urban	*p*-Value	Primary	Mixed	Secondary	*p*-Value
Crosswalks not at intersections (No. of schools with Google Street View)	91	2	39	17	33		48	16	27	
Low quality, %	17.6	50.0	7.7	7.4	33.3	0.19	18.8	0	25.9	0.10
Moderate quality, %	25.3	0	30.8	14.8	21.2		22.9	18.8	33.3	
High quality, %	57.1	50.0	61.5	40.7	48.5		58.3	81.2	40.7	
Crosswalks at intersections (No. of schools with Google Street View)	264	6	93	56	109		124	55	85	
Low quality, %	30.7	33.3	24.7	17.9	42.2	<0.001	40.3	29.1	35.5	0.36
Moderate quality, %	33.3	33.3	22.6	44.6	36.7		31.5	30.9	37.6	
High quality, %	36.0	33.3	52.7	37.5	21.1		28.2	40.0	27.1	
Traffic medians (No. of schools with Google Street View)	98	1	27	21	49		35	25	38	
Not suitable for refuge, %	11.2	0	0	5.8	2.2	0.60	12.2	12.5	5.3	0.08
Suitable for refuge, %	88.8	100	100	84.2	87.8		87.8	87.5	94.7	
Sidewalk conditions (No. of schools with Google Street View)	271	6	92	54	116		127	51	90	
Low quality, %	1.1	0	1.9	0	0.9	0.65	0.8	1.9	1.1	0.82
High quality, %	98.9	100	97.9	100	99.1		99.2	98.1	99.9	
Bicycle lanes (No. of schools with Google Street View)	32	0	11	9	12		12	11	9	
Separated by painted line, %	81.3	0	63.6	77.8	100	0.08	83.3	81.1	77.8	0.95
Separated by physical barrier or space, %	18.8	0	36.4	22.2	0		16.7	18.2	22.2	

**Table 3:** Perceived traffic problems in the neighbourhoods surrounding Canadian schools.

	Total	Urban/Rural Location of School					Type of School			
		Rural	Small	Medium	Urban	*p*-Value	Primary	Mixed	Secondary	*p*-Value
Traffic problems (No. of schools with administrative questionnaire)	399	51	174	60	114		180	108	111	
Not a problem, %	52.4	84.0	63.0	36.7	29.8	<0.001	52.2	61.1	44.1	0.08
Minor problem, %	30.1	12.0	24.3	41.7	41.2		33.3	23.1	31.5	
Moderate problem, %	13.5	4.0	9.8	18.3	21.1		12.2	12.0	17.1	
Major problem, %	4.0	0	2.9	3.3	7.9		2.2	3.7	7.2	

Only 10% of the Canadian schools under study had roads with a designated bicycle lane. Multiple case studies have shown bicycle-specific facilities are associated with the uptake of active transportation [4,5] and injury reduction in cyclists [24,25,26]. This provides an opportunity to incorporate cycling infrastructure (either on-road marked lanes or segregated lanes) into the built environment. Purposeful bicycle-friendly roadway design coupled with cycling safety education for children are recommended for bicycling promotion and injury prevention. Most bicycle lanes were separated from traffic by a painted line which may appeal to a smaller group of users and be less safe than if separated by a physical barrier [27]. The latter policy recommendation, while optimal, is made while recognizing that there are practical limits for infrastructure improvement in some communities that rely mainly on municipal and other government funding and a limited tax base.

Studies in the United Kingdom, Australia, and Sweden have shown that reducing speeds in areas comparable to schools zones with a high amount of road users can be effective in reducing road traffic injury [28]. Thus, as a policy solution, incorporating traffic calming measures such as speed bumps or chokers into the road design around schools can have a positive impact on related injury rates [29]. Although helpful for injury prevention, an important consideration for policy makers is that the installation of speed bumps can be contentious in Canadian communities due to concerns about speed control for regular driving, impeding emergency vehicles, and snow removal [30]. This has been remedied by following specific modern design criteria and avoiding installation on busy transit routes. These measures have been shown to result in negligible delays to maintenance and service vehicles [31].

Public health efforts to foster active transportation and prevent injury are especially needed to promote safe school road environments in rural areas [32]. Construction of sidewalks, crosswalks, and traffic medians to prevent pedestrian and cyclist injury and facilitate active transportation in the rural environment would, theoretically, be the optimal solution to these inequities. However, there may be minimal resources to complete large-scale projects of this nature, and they will require public commitment and planning. Other challenges to infrastructure improvement in rural areas include those surrounding road types (highways that are not amenable to pedestrian accommodations) or distance across the road (narrow roads may not be wide enough to support a traffic median). Novel integration of both types of initiatives is required to establish the desired outcome of children walking or bicycling to school without risk for injury, especially in rural areas.

Consistent with their setting, primary schools were more likely to have narrower roads in front of the school but less likely to have full sidewalk coverage on the school’s surrounding roads. This may represent an important inconsistency. Narrow road lanes can lower traffic speeds [33] and sidewalk coverage is favorable for pedestrian safety [34]. Adding longitudinal pavement markings that visually indicate narrower roads may be an effective and relatively inexpensive intervention to reduce traffic speeds [33] and potentially remedy pedestrian safety concerns and injuries around secondary schools.

The quality of safety features around Canadian schools is varied. When present, sidewalks are generally of high quality with minimal cracks and surface defects. Most of the traffic medians that were assessed were suitable for pedestrian refuge. Crosswalk quality should be maintained around schools especially in urban areas where traffic is of the highest concern. Since 30% of the crosswalks at intersections around Canadian schools were rated as being of low quality, a relatively inexpensive intervention could be re-painting over the lines to enhance visibility. Increased pedestrian risk-taking behavior has sometimes been noted in the presence of safer environments [35], although such “risk homeostasis” theories are controversial. Ages of neighbourhoods may also be a factor influencing the quality of safety features such as painted crosswalks and bicycle lanes, sidewalk condition, or even sidewalk presence due to weathering effects of deterioration over time. The accessed Google images acquired in the early spring were less favourable for analysis due to municipal road painting schedules which may have been a factor in quality measures.

Strengths of this study are size and national scope, as well as its novelty to the built environment and (indirectly) population health literatures in Canada. The basic descriptive information provides a starting point for understanding road features that promote or hinder safety of children as they commute to school, and this in itself is valuable for policy and prevention efforts. Limitations include the fact that responses to the administrator survey represent subjective opinions. Two provinces (New Brunswick and Prince Edward Island) were unable to participate in the 2009/2010 Cycle of HBSC, which limits the representativeness of the sample. Data were unavailable for some measures due to limited coverage of imaging in rural and remote areas [36]. Some contained satellite imaging but lacked street view imaging, and some had no images available. We were also unable to examine road condition or quality in the absence of Street View measures. Another limitation was our protocol for Google Earth street view assessment. We analyzed the safety features of the roads immediately surrounding the school property and there were instances where safety features were not found on the roads we choose to capture but were likely present on the roads adjacent to the immediate surrounding roads. Thus, lack of safety features on adjacent roads to the school properties should not necessarily be interpreted as a lack of safety features within the entire school active transportation environment.

Finally, our research suggests the need to investigate active transportation patterns and associated injury risks surrounding Canadian schools in relation to the active transportation safety environment. This would benefit policymakers to determine which areas are most appropriate for intervention. National initiatives such as Canada’s Road Safety Strategy (RSS) 2015 [37,38] could benefit from our study findings. Furthermore, there is a lack of available information about the safety environments surrounding schools internationally. Therefore, this study could serve as a possible framework for how to complete comprehensive analysis for other countries involved in like prevention efforts. Our results revealed specific areas in which Canadian schools are lacking pedestrian/cyclist infrastructure and road safety features compared to the very small amount of international research based on identifying these features surrounding schools. We therefore believe that this study is pertinent in its contribution to the literature surrounding active transportation and safety environments internationally.
